# Clinical evaluation of autistic symptoms in women with anorexia nervosa

**DOI:** 10.1186/s13229-017-0128-x

**Published:** 2017-03-16

**Authors:** Heather Westwood, William Mandy, Kate Tchanturia

**Affiliations:** 1 Psychological Medicine, Institute of Psychiatry, Psychology and Neuroscience, King’s College London, London, UK; 2 Research Department of Clinical, Educational and Health Psychology, University College London, 103 Denmark Hill, London, SE5 8AF UK; 3 Ilia State University, Tbilisi, Georgia; 4 Psychological Medicine Clinical Academic Group, South London and Maudsley NHS Foundation Trust National Eating Disorders Service, London, UK

**Keywords:** Anorexia nervosa, Eating disorder, Autism spectrum disorder, ADOS-2, Female autism

## Abstract

**Background:**

Despite a suggested link between anorexia nervosa (AN) and autism spectrum disorder (ASD), previous studies have used self-report or diagnostic criteria to assess for ASD in AN populations, rather than direct observation of symptom characteristic of ASD. The aim of this study was to use a standardised, clinical assessment of ASD, the Autism Diagnostic Observation Schedule, 2nd Edition (ADOS-2), to investigate the presence of autistic symptoms in a cross-sectional sample of women with AN.

**Methods:**

Sixty women were recruited from inpatient or day-patient specialist eating disorder services. Each participant underwent the ADOS-2 assessment and completed a set of self-report questionnaires assessing eating disorder pathology and other psychiatric symptoms. IQ was also assessed.

**Results:**

Fourteen women (23.3%) scored above clinical cutoff for ASD on the ADOS-2. Only eight of these women displayed repetitive or restrictive behaviours, while all 14 had difficulties with social affect. Elevated ASD symptoms were associated with increased alexithymia and obsessive-compulsive symptoms, but not specific eating disorder pathology.

**Conclusions:**

ASD symptoms are over-represented in women with severe AN and appear to be associated with other psychiatric symptoms, which warrant further investigation and consideration in treatment.

## Background

Anorexia nervosa (AN) is a severe eating disorder (ED) characterised by low body weight, restriction of energy intake relative to requirements and undue influence of weight and shape on self-evaluation [1]. The disorder tends to be diagnosed during adolescence [2, 3] and affects significantly more females than males [4, 5]. In contrast, autism spectrum disorder (ASD) is a developmental disorder defined by difficulties with social interaction, communication and repetitive and restricted behaviours (RRBs) [1]. ASD affects approximately 1% of the population, 0.2% of whom are female [6]. It is considered a heterogenic, dimensional disorder with the identification of subgroups, based on the presence of co-occurring psychiatric, medical and/or genetic risk of continuing importance to research and clinical practice [7].

While both ASD and AN are rare disorders in the general population, ASD appears to be over-represented within ED populations, with a systematic review reporting a mean prevalence of 22.9% [8]. Much of the research on ASD in AN has come from a Swedish cohort who was assessed for ASD at regular intervals over a 16-year period [9]. In each of the four assessments, the diagnostic tools used to assess ASD differed, yielding differing rates of ASD diagnosis. This underlines the difficulty of assessing for ASD in AN, particularly in light of evolving diagnostic criteria and the presence of a distinct female autism phenotype [10], which makes accurately assessing the disorder in females challenging [11]. There is also a possibility that the acute, starved state associated with AN may exacerbate the presence of symptoms characteristic of ASD [12]. This state versus trait argument [12] has led to calls for further research to disentangle the complex relationship between AN and ASD.

One of the criticisms of research examining the presence of ASD in AN is the use of inconsistent screening tools and varying diagnostic criteria [8, 13], instead of ‘gold-standard’ clinical assessment. More recently, studies in younger people, utilising parental-report methods [14, 15], have reported the prevalence rate of ASD to be substantially lower than in adult studies. While this is consistent with the idea that AN may exacerbate the presence of ASD symptoms, diagnostic tools which rely on parental report rather than direct observations of autistic behaviour may result in under-reporting of ASD, particularly in a predominantly female sample, in which ASD may not be recognised.

Females with ASD display less RRBs than their male counterparts and may be able to mask their social difficulties, leading to under-recognition or missed diagnoses [16, 17]. The high likelihood that ASD will remain undetected in females [18] can cause stress and anxiety and may lead to the high proportion of females with ASD whom experience co-morbid mental health difficulties [17, 19]. This applies not only to ASD but also to elevated symptoms of autism, which extend throughout the population [20]. Thus, the presence of ASD symptoms may act as a risk factor for a range of emotional and behaviour issues [21], including eating problems. It stands to reason that females with elevated ASD symptoms may be vulnerable to the development of secondary mental health problems and are thus likely to be over-represented in ED populations. Alternatively, the gender bias in diagnosis may result in females with ASD being mislabelled as having an ED, particularly as features of AN such as extreme rigidity in eating or obsession with exercise may mask the presence of ASD [13, 22].

Despite ASD presenting differently in females, gender-specific diagnostic tools and criteria are yet to be widely implemented [23]. As such, the diagnosis of adults with ASD relies on standardised assessment tools including the Autism Diagnostic Observation Schedule, 2nd Edition (ADOS-2; [24]) which is yet to be tested with women with AN. In a small-scale feasibility study [13], the ADOS-2 was used with a pre-selected sample of women with AN who presented with social and flexibility difficulties. Of ten women assessed, half received an ADOS-2 ASD classification. This study demonstrated the feasibility of using the ADOS-2 with women with AN. However, as the study used a small sample, pre-selected for having a suspected ASD, the high prevalence of observed autistic difficulties cannot be generalised to the wider AN population.

The ADOS-2 has been criticised for not showing adequate specificity and sensitivity to discriminate adults with comorbidities and no previous diagnosis of ASD [16, 25], leading to the use of alternative measures such as the Ritvo Autism Asperger Diagnostic Scale, Revised (RAADS-R; [26]), with people with EDs [27]. Using the RAADS-R, 33% of participants were classified as having elevated ASD traits. However, the RAADS-R relies on self-report whereas the recommended diagnosis of ASD in adults should incorporate both self-report and observational measures [28]. While observational measures should not be used in isolation and may not be necessary for a diagnosis of ASD to be given [29], the ADOS-2 is nevertheless a widely used measure of ASD symptoms. Studies utilising this tool in AN populations will therefore provide a useful comparison to other studies, such as those utilising self-report measures to assess for ASD traits [30].

This challenge of diagnosing ASD in AN is exacerbated by the high level of comorbidities in EDs, which may mediate the relationship between ASD and AN. Up to 97% of individuals hospitalised for AN are thought to have at least one psychiatric comorbidity [31], with particularly high rates of depression [32] and anxiety disorders including obsessive-compulsive disorder (OCD) [33, 34]. The presence of such comorbidities along with the possibility that starvation exacerbates ASD symptoms [35] confounds the ability to differentiate between these disorders.

### Aims

The primary aim of the current study was to examine the presence of autistic symptoms, as measured via direct observation using the ADOS-2, in women hospitalised for AN. In addition, the study aimed to explore group differences in symptoms of eating pathology as well as other psychiatric symptoms of depression, anxiety, alexithymia and OCD between individuals with high, sub-clinical and no symptoms of ASD.

## Methods

### Study design and participants

A cross-sectional design was used to assess the presence of ASD symptoms within a subset of women receiving either inpatient or day-patient treatment for AN. Participants were recruited from three specialist ED services and had a primary diagnosis of AN according to the Diagnostic and Statistical Manual of Mental Disorders, 5th edition eating disorder (DSM-5; [1]) made prior to admission to the recruitment sites, although exact diagnostic procedures may have varied across tertiary services. Inclusion criteria for the study were the following: (a) aged between 18 and 55 years, (b) no diagnosed history of a neurological condition or acquired brain injury, (c) English speaking, (d) female and (e) ability and willingness to provide informed written consent to participate. A total of 60 women with an age range of 18 to 47 were recruited over 14 months. Fifty-one participants were recruited from the same national ED service, constituting 68% of admissions to that service over the recruitment period. The remaining participants were recruited from another inpatient service (*N* = 6) or day care (*N* = 3). The study was reviewed and approved by the National Research Ethics Service (14/LO/2131)

### Procedure

Each participant completed the ADOS-2, and clinical information was obtained from patient records on current body mass index (BMI) and illness duration, defined as the number of years since the participant was first diagnosed with AN. In addition, participants were asked to complete a set of self-report questionnaires assessing for the presence of depression, anxiety, alexithymia and obsessive compulsive symptoms. All assessments were conducted by the first author, certified research-reliable in the administration and scoring of the ADOS-2, and were video recorded as per procedural recommendations [24].

### Measures

#### Autism Diagnostic Observation Schedule, 2nd Edition (ADOS-2; [24])

The ADOS-2 is the most widely used and best validated direct observation of characteristics associated with ASD [28]. It focuses on the domains of social interaction, communication, play and imaginative use of materials and takes approximately 40 min to administer. The ADOS-2 has four modules, one of which is selected for the participant, dependent upon their expressive language ability. Module 4 is designed for use with verbally fluent adolescents and adults and was used for all participants in the current study. The assessment is scored according to a standardised system and diagnostic algorithm. Scores from the ‘Stereotyped Behaviours and Restricted Interests’ are not included in the diagnostic classification as these are low-frequency behaviours which may not present in a time-limed direct observation.

The Module 4 algorithm [36] has recently been revised to increase comparability of Module 4 to other ADOS-2 modules and to map on to updated DSM-5 diagnostic criteria. The new algorithm consists of two sub-scores: Social Affect and RRBs. For the diagnostic cutoff, only the Social Affect and the sum of the two subscales are used. With respect to clinician ASD diagnosis, the revised Module 4 algorithm demonstrated high sensitivity (90.5%) and specificity (82.2%). The revised algorithm is superior to the original in that it has greater sensitivity and better reflects the current diagnostic criteria, particularly the symptoms displayed by females and adults with ASD (DSM-5) [37]. For the purposes of this study, each participant was scored using both the original and revised algorithms for comparison with other published studies.

#### Eating Disorder Examination Questionnaire (EDE-Q; [38])

The EDE-Q is a standardised and well-validated self-report measure of the severity of the characteristic psychopathology of EDs. Respondents are asked to rate how often they have engaged in certain eating disordered behaviours or held eating disordered concerns over the past 28 days. The scores result in a ‘global’ or total score and four subscale scores: ‘eating concern’, ‘weight concern’, ‘shape concern’ and ‘restraint’. The maximum global score is 6, with higher scores indicating greater severity. The optimal cutoff score to discriminate between females with the disorder and healthy controls is 2.5 [39]. In this study, Cronbach’s alpha was .83, indicating good internal reliability.

#### Hospital Anxiety and Depression Scale (HADS; [40])

The HADS is a widely used 14-item self-rating instrument for anxiety and depression in patients with both physical and mental health problems. The maximum possible score on either subscale (anxiety/depression) is 21 with a clinical cutoff of 10. For this study, Cronbach’s alpha was .90.

#### The 20-item Toronto Alexithymia Scale (TAS-20; [41])

The TAS-20 is a validated measure of alexithymia (the inability to label and describe emotions in the self) with good internal consistency and test-retest reliability. It is a 20-item self-report measure with three subscales: difficulty describing feelings; difficulty identifying feelings and externally orientated thinking. A clinical cutoff of 61 is indicative of high alexithymia [42]; Cronbach’s alpha was .77 in this study, indicating acceptable internal reliability.

#### The Obsessive-Compulsive Inventory, Revised (OCI-R; [43])

The OCI-R is an 18-item self-report scale with a five-point Likert scale format. It consists of six subscales: checking, washing, obsessing, neutralising, ordering and hoarding, yielding both a total and individual subscale scores. The total score ranges from 0 to 72 with a suggested cutoff of 21 for distinguishing patients with OCD from non-anxious controls [43]. The OCI-R has good to excellent internal reliability among both clinically anxious and non-anxious participants and good test-retest reliability. For this study, Cronbach’s alpha was .94.

#### Wechsler Abbreviated Scale of Intelligence—2nd Edition (WASI-II; [44])

The WASI-II is an assessment of intelligence, suitable for individuals aged 6–90 years old. It provides estimate of verbal and perceptual reasoning as well as full-scale IQ.

### Statistical analysis

Presence of ASD symptoms was assessed by calculating the percentage of participants who scored above the suggested clinical cutoff on the original and revised diagnostic algorithms of the ADOS-2. To explore clinical difficulties across the range of autistic symptom severity, participants were split into three groups: individuals who scored above clinical cutoff on the ADOS-2, representing high levels of autistic symptoms (HAS); individuals with sub-clinical autistic symptoms (SCAS) and those who scored zero on the ADOS-2 (NAS). The decision to split participants into these groups, rather than to analyse ADOS-2 scores as a continuous variable, reflected the non-normal distribution of ADOS-2 scores. Specifically, this distribution showed a floor effect of the ADOS-2 with a substantial proportion (*N* = 22) of the sample scoring zero on its diagnostic algorithm.

Distribution of the clinical symptom scores was assessed visually using Q-Q plots and statistically using the Shapiro-Wilk test. As age, illness duration, full-scale IQ and the scores on the EDE-Q were not normally distributed (*p* < .05) across all ASD groups, Kruskal-Wallis tests were used to analyse group differences in these scores. If significant differences were indicated, post hoc analysis using Mann-Whitney tests was used to determine where the difference was. To account for multiple comparisons (three comparisons per dependent variable), the Bonferroni correction was applied so all effects are reported at a .0167 level of significance. Pearson’s *r* effect sizes are provided for all significant group differences for non-parametric analysis.

One-way ANOVAs were used to explore group differences in BMI, depression, anxiety, alexithymia and obsessive-compulsive symptoms. For ANOVA analyses, omega squared (ω^2^) effect size was calculated using the following interpretation: small (.01), medium (.06) and large (.14) [45]. If results of the ANOVAs were statistically significant, Tukey’s post hoc tests were used to determine where the differences were. As some participants did not complete all questionnaires, i.e. out of choice or due to missing items, the *N* for each measure is indicated in the results section. All data were analysed using the statistical package IBM SPSS version 22.00.

## Results

A comparison of the sociodemographic and clinical characteristics of the participant groups is displayed in Table 1.

**Table 1:** Sociodemographic and clinical comparison of HAS, SCAS and NAS groups

	HAS mean (SD)	SCAS mean (SD)	NAS mean (SD)	Test statistic	*p*	Effect size
Age^a^	*N* = 14	*N* = 24	*N* = 22			
	26.5	23	22	*H*(2) = 1.12	.572	
EDE-Q^a^	*N* = 14	*N* = 24	*N* = 22			
Global	4.65	4.56	3.83	*H*(2) = 4.13	.123	
Eating concern	3.63	4.25	3.36	*H*(2) = 1.56	.450	
Shape concern	5.5	5.5	4.44	*H*(2) = 2.85	.241	
Weight concern	4.8	4.8	3.7	*H*(2) = 5.59	.061	
Restraint	4.4	3.8	2.9	*H*(2) = 4.74	.093	
ED duration (years) ^a^	*N* = 14	*N* = 24	*N* = 22			
	7.5	6.5	5	*H*(2) = 1.18	.555	
BMI	*N* = 14	*N* = 24	*N* = 21			
	15.51 (2.19)	15.8 (2.02)	14.41 (2.09)	*F*(2,56) = 1.61	.209	*ω2* = .02.
HADS depression	*N* = 14	*N* = 24	*N* = 22			
	12.29 (5.01)	9.46 (4.83)	8.41 (4.27)	*F*(2, 57) = 2.99	.058	*ω2* = .06
HADS anxiety	*N* = 14	*N* = 24	*N* = 22			
	15.79 (4.44)	14.42 (4.58)	11.91 (5.54)	*F*(2,57) = 2.95	.06	*ω2* = .06
TAS-20	*N* = 14	*N* = 23	*N* = 22			
Total	69.5 (7.67)	62.26 (12.17)	59.18 (11.02)	*F*(2,56) = 3.93	*.025*	*ω2* = .09
Describing feelings	18.57 (2.71)	16.16 (3.12)	14.36 (4.19)	*F*(2,56) = 6.18	*.004*	*ω2* = .15
Identifying feelings	23 (4.80)	18 (6.67)	16.05 (5.69)	*F*(2,56) = 6.01	*.004*	*ω2* = .15
Externally oriented	24 (3.01)	24.91 (3.75)	25.77 (2.99)	*F*(2,56) = 1.24	.298	*ω2* = .01
OCI-R	*N* = 12	*N* = 22	*N* = 21			
	41.92 (18.16)	33.41 (18.10)	22.19 (12.30)	*F*(2,52) = 6.14	*.004*	*ω2* = .16
WASI-II full-scale IQ^a^	*N* = 11	*N* = 22	*N* = 20			
	101	107.5	117	*H*(2) = 1.75	.417	

Significant differences are set in italics

^a^Medians are displayed, analysed using the non-parametric Kruskal-Wallis test

### Presence of elevated ASD symptoms

**Fig. 1:**
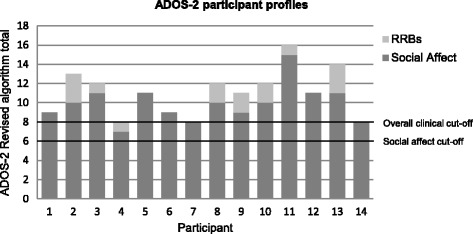
ADOS-2 scores for participants scoring above diagnostic cutoff (HAS group). Each *bar* represents the total algorithm score for each participant, consisting of the total social affect and RRB subscale scores

Of the 60 women assessed, 13 scored above clinical cutoff for ASD on the ADOS-2 according to the original, standardised algorithm [46]. Applying the revised algorithm, [36] 14 participants (23.3%) scored above cutoff. The discrepancy between the original and revised algorithms for this individual was due to a low score on the ‘Communication’ subscale on the original algorithm. Figure 1 shows the scoring profiles for the 14 participants who scored above clinical cutoff on the revised algorithm.

### Specific ADOS-2 profile of AN sample

Only eight women who scored above the clinical cutoff for ASD presented with RRBs. The nature of these RRBs are shown in Table 2. A comparison of the scores of our AN sample with previously published Module 4 scores are displayed in Table 3.

**Table 2:** Frequency of repetitive and restricted behaviours recorded in the HAS group during the ADOS-2

RRB	Frequency of behaviour in HAS group
Speech abnormalities associated with autism	7
Stereotyped/idiosyncratic use of words or phrases	3
Unusual sensory interest in play material/person	0
Excessive interest in or reference to unusual or highly specific topics or objects or repetitive behaviours	4
Hand and finger and other complex mannerisms	0

**Table 3:** Comparison of ADOS-2 scores with published scores from Pugliese et al. [37]

		HAS	Published ASD	SCAS	Published non-ASD
*N*		14	253	24	68
Original	Comm.	2.92 (0.64)	3.85 (1.58)^a^	1.21 (1.02)	1.32 (1.34)^a^
Algorithm	Social interaction	7.08 (1.85)	8.38 (2.63)^a^	2.33 (1.83)	3.09 (2.50)^a^
Mean (SD)	Comm. + social interaction	10.0 (2.04)	11.23 (4.42)	3.54 (2.04)	5.34 (4.47)
Revised	Social affect	9.93 (1.94)	9.96 (4.58)	3.46 (2.04)	4.69 (4.54)
Algorithm	RRBs	1.07 (1.14)	2.76 (1.84)	0.37 (0.58)	1.24 (1.53)
Mean(SD)	Social affect + RRBs	11.0 (2.42)	N/R	3.83 (2.14)	N/R

Individuals with AN who scored 0 on the ADOS-2 (NAS) have been excluded

*N/R* not reported

^a^From: Hus and Lord [36]

### Relationship between ADOS-2 scores and clinical symptoms

#### Alexithymia

Tukey’s post hoc analysis revealed that the increase in TAS-20 total scores from NAS to SCAS (3.08, 95% CI (−4.7 to 10.86)) was not statistically significant, *p* = .610, nor was the increase from SCAS to HAS (7.24, 95% CI [−1.61 to 16.08], *p* = .129). However, there was a significant increase in score from the NAS to the HAS group (10.32, 95% CI [1.4 to 19.24], *p* = .020).

For the TAS-20 ‘difficulty describing feelings’ subscale, the increase in scores from NAS to SCAS groups (1.81, 95% CI [−.71 to 4.33]) was not statistically significant (*p* = .202), nor was the increase from SCAS to HAS groups (2.4, 95% CI [−.46 to 5.26], *p* = .117). The difference in score between the NAS and HAS groups (4.21, 95% CI [1.32 to 7.09]) was statistically significant (*p* = .003).

On the ‘difficulty identifying feelings’ subscale, the increase in scores from the NAS to SCAS group (1.96, 95% CI [−2.30 to 6.21]) was not significant (*p* = .514). However, there were significant increases in scores between the SCAS and HAS groups (5, 95% CI [.17 to 9.83], *p* = .041) and between the NAS and HAS groups (6.96, 95% CI [2.08 to 11.83], *p* = .003).

#### Obsessive-compulsive symptoms

Tukey’s post hoc analysis revealed that the only significant increase in OCI-R scores was from the NAS to the HAS groups (19.73, 95% CI [5.66 to 33.79], *p* = .004).

## Discussion

This study is the first to examine the presence of observable ASD symptoms in a cross-sectional sample of adult women hospitalised for AN, using the ADOS-2. Of the 60 women assessed, 14 (23.3%) scored above clinical cutoff on the revised algorithm, indicating the presence of clinical symptoms associated with ASD. The finding is in line with previous prevalence estimates in adults with EDs, in particular from Huke et al.’s [8] systematic review, which reported an estimated average prevalence rate of 22.9%. However, the rate is higher than in studies with young people, which have suggested only a slightly increased prevalence of 4% [14, 15]. As these studies did not use observational measures of ASD symptoms, the results are not directly comparable.

Using the revised diagnostic algorithm [36], all 14 women who scored above clinical cutoff displayed difficulties with social affect, but notably, only eight displayed RRBs. While RRBs are not included in the ADOS-2 diagnostic algorithm, they form part of the diagnostic criteria for ASD [1]. This level of RRBs is also lower than in adults with ASD without AN, reported in one previously published study using the revised algorithm [37]. The most common RRB displayed in the HAS group was speech abnormalities associated with ASD which includes unusual intonation, volume, rhythm or rate. There was no evidence of sensory interests or hand mannerisms in any participant.

Females with ASD have been found to display less RRBs than males [10], and RRBs are less common in adults compared with younger individuals [47]. Therefore, it may be that the women who scored above cutoff represent the typical, under-recognised profile of ASD often seen in high-functioning females without intellectual disability [48], particularly as AN is characterised by above average intelligence (for systematic review: [49]). In addition, it could be that the ADOS-2 was not sensitive enough to detect RRBs in females with AN. RRBs are notoriously hard to measure using direct observation, which is why they are not included in the ADOS-2 diagnostic algorithm [24]. Therefore, they may have been present in the participants in this study but were not detected during assessment.

There is also a possibility that the RRBs displayed in participants in this study are not manifestations of ASD but are instead associated with other psychiatric symptoms including depression or anxiety. This could account for the flat intonation displayed by several participants and explain why other stereotypies associated with ASD, including unusual sensory interests or complex hand mannerisms, were not observed in any participants. In fact, the observed difficulties with social affect could also occur within the context of anxiety and depressive symptoms and are thus not necessarily indicative of ASD. Delineating differential diagnoses, overlapping symptoms and true comorbidities remains a key clinical challenge [22], not only in the field of AN but also in the accurate diagnosis of ASD in all clinical populations.

Despite these findings supporting the idea of an overlap between AN and ASD, the aetiology of these symptoms remains unclear. Observational measures such as the ADOS-2 cannot determine the extent to which ASD is truly over-represented in AN or whether the observed symptoms, at least in some cases, represent epiphenomena, secondary to the ED. In this study, elevated ASD symptoms were not related to ED psychopathology, BMI or illness duration. These findings appear to be contrary to the suggestion of ASD symptoms arising from the starved state of AN [12] as if this was the case, one would predict a linear association between ASD symptoms and ED symptoms. However, other symptoms of AN including lowest recorded BMI or the presence of amenorrhea were not examined in this study, and future research would benefit from examining the association between ED severity and ASD more closely, using a longitudinal design.

This study also suggests that in AN, the presence of elevated ASD symptoms is associated with the presence of alexithymia and obsessive-compulsive symptoms. While it is possible that symptoms such as these may mediate the relationship between AN and ASD, causing individuals to appear autistic as a secondary effect of other symptoms, it is also possible that the presence of ASD symptoms in females may indirectly cause additional mental health problems for a range of reasons, including the stress and anxiety caused by attempting to mask social difficulties [22].

While disorders such as OCD are common in both AN and ASD separately [31, 50], it may also be that the presence of both AN and elevated ASD symptoms increases the likelihood of other co-occurring psychiatric symptoms. Individuals with both diagnosed ASD and OCD score higher on the OCI-R than those with ASD alone [51], and as these disorders are not mutually exclusive, it may be that this pattern is the same in individuals with AN. Additionally, both AN and ASD are associated with increased alexithymia [52], with its presence contributing to poorer treatment outcome in AN [53] and therefore the association between ASD symptoms and alexithymia in AN is worthy of consideration.

Psychiatric symptoms such as alexithymia may also compound the social difficulties and isolation of individuals with AN [54], thus causing them to score highly on the ADOS-2, without having a ‘true’ ASD. While research on the use of the ADOS-2 in complex psychiatric groups is limited, overlap in symptoms between disorders may lead to false-positive results using its current diagnostic algorithm. A study using the ADOS and the Autism Diagnostic Interview, Revised (ADI-R; [55]), in children with psychosis found that participants met criteria for ASD on this measure despite not receiving a clinical diagnosis from clinicians [56]. Standard diagnostic assessment packages, such as the ADOS/ADI-R, may therefore have limited use, particularly for clinically complex groups, highlighting the need for robust clinical assessment.

The cross-sectional design of the current study and the small number of participants who scored above clinical cutoff for ASD limit analysis to the statistical associations between variables, preventing examination of predictor variables or causal relationships to be made. This is the main limitation of this study, and the underlying aetiology of ASD symptoms in AN remains inconclusive. In addition, the assessment of ASD was limited to a single observation of symptoms. While the ADOS-2 is a highly specific and sensitive assessment tool, clinical diagnosis of ASD is multi-model, utilising direct observation, self-report, developmental history and occupational information. Future longitudinal studies using both the ADOS-2 and a standardised parental report measure are needed to confirm the precise nature of the relationship between ASD and AN. As all participants in this study were recruited from inpatient or day-patient services, they are likely to have more severe or complex eating disorder pathology. Therefore, the findings may not translate to other patient groups, such as individuals receiving less intensive treatment. It is possible that this specific patient group experience higher levels of comorbidity, and thus, future studies examining ASD symptoms across the spectrum of EDs are warranted.

## Conclusions

This study is the first to use a gold-standard observational assessment to examine the presence of ASD symptoms in women with severe AN. 23.3% scored above the suggested clinical cutoff on the ADOS-2, suggesting the presence of elevated ASD symptoms. These symptoms appeared to be associated with other psychiatric symptoms but not specific ED pathology. Autistic symptoms co-occur with a range of mental health problems which warrant further investigation and consideration in treatment.

## Abbreviations

ADOS-2: Autism Diagnostic Observation Schedule, 2nd Edition
AN: Anorexia nervosa
ASD: Autism spectrum disorder
BMI: Body mass index
DSM-5: Diagnostic and Statistical Manual of Mental Disorders, 5th Edition
ED: Eating disorder
EDE-Q: Eating Disorder Examination Questionnaire
HADS: Hospital Anxiety and Depression Scale
HAS: High autistic symptoms
IQ: Intelligence quotient
NAS: No autistic symptoms
OCD: Obsessive-compulsive disorder
OCI-R: Obsessive-Compulsive Inventory, Revised
RAADS-R: Ritvo Autism Asperger Diagnostic Scale, Revised
RRBs: Repetitive and restricted behaviours
SCAS: Sub-clinical autistic symptoms
TAS-20: Toronto Alexithymia Scale, 20-item version
WASI-II: Wechsler Abbreviated Scale of Intelligence, 2nd Edition
